# Editorial: Orofacial pain, bruxism, and sleep, volume II

**DOI:** 10.3389/fneur.2023.1331275

**Published:** 2023-11-21

**Authors:** Mieszko Wieckiewicz, Ephraim Winocur

**Affiliations:** ^1^Department of Experimental Dentistry, Wroclaw Medical University, Wrocław, Poland; ^2^Department of Oral Rehabilitation, Maurice and Gabriela School of Dental Medicine, Tel Aviv University, Tel Aviv, Israel

**Keywords:** orofacial pain, bruxism, sleep, obstructive sleep apnea, headache

According to the revised definition by the International Association for the Study of Pain (IASP), pain is now defined as “an unpleasant sensory and emotional experience associated with actual or potential tissue damage, or one that resembles such an experience” (1). Orofacial pain, on the other hand, is described as “a common type of pain perceived in the face and/or oral cavity, resulting from diseases or disorders of regional structures, dysfunction of the nervous system, or from referral from distant sources” (2).

The current international consensus (3) divides bruxism into two circadian manifestations: awake bruxism (AB) and sleep bruxism (SB). AB is “masticatory muscle activity during wakefulness characterized by repetitive or sustained tooth contact and/or bracing or thrusting of the mandible and is not considered a movement disorder in otherwise healthy individuals.” SB is “masticatory muscle activity during sleep characterized as either rhythmic (phasic) or non-rhythmic (tonic) and is not deemed a movement or sleep disorder in otherwise healthy individuals.” Bruxism is now viewed as a behavior with a central origin and genetic contribution, rather than a parafunction, disorder, or disease in otherwise healthy individuals (3, 4).

Sleep can be defined as “a physiological state characterized by alterations in brain wave activity, breathing, heart rate, body temperature, muscle function, and other bodily functions” (5).

Orofacial pain, bruxism, and sleep disorders are interconnected. Complex cause-and-effect relationships exist between them, as does co-occurrence (6–16). These conditions are found in both genders and across all age groups, highlighting their widespread prevalence (17). They can also arise or intensify due to various internal and external factors, e.g., a pandemic or war and/or an impaired lifestyle, psychoemotional disorders, chronic stress, ineffective stress coping mechanisms, addictions, genetic predispositions, and more (18–23). Numerous evidence-based methods are available to address and manage these issues (24–29).

Given the intricacies of the topics discussed (etiology, prevalence, cause and effect relationships, co-occurrence, diagnostics, and management), we urge readers to delve deeper into subjects like the traffic-light prognosis-based classification system for managing patients with headache and/or orofacial pain in musculoskeletal practice, the relationship between sleep bruxism and obstructive sleep apnea in relation to sleep architecture, a systematic review of current scientific literature evaluating masticatory muscle activity in individuals with pain-related temporomandibular disorders using surface electromyography, and the dynamic changes in local metrics for patients experiencing toothaches in a resting state, to enhance understanding of the central neural mechanisms in patients with dental pain and its cognitive and emotional effects (Wang et al.; Greenbaum and Emodi-Perlman; Okura et al.; Szyszka-Sommerfeld et al.).

## Author contributions

MW: Conceptualization, Writing—original draft, Writing—review & editing. EW: Conceptualization, Writing—original draft, Writing—review & editing.
